# Supplementary material for: Analysis of Domestication Loci in Wild Rice Populations
Source: Plants (Basel). 2023 Jan 20;12(3):489. doi: 10.3390/plants12030489 (PMC9919211; doi:10.3390/plants12030489)
Supplement: Supplementary file 1 [file plants-12-00489-s001.zip › plants-1952851-supplementary.pdf]

Table S1. Brief statistics of whole-genome sequencing of 26 wild rice samples and mapping outputs of 26 samples reads to *O. sativa* ssp. *japonica* cv. Nipponbare as a reference genome. This data is derived from Hasan et al. [34].

Wild rice accession	Total number of trimmed reads	Total number of trimmed nucleotides	Average sequence read length (times reference length)	Average mapped sequence read depth (times reference length)	Mapped reads (%)	Mapped bases (%)	Total consensus length (bp)	Total consensus as % of reference
WR24	98,924,768	14,260,593,039	37.32	8.88	23.98	23.79	322,074,565	81.96
WR44	79,543,302	11,329,429,070	29.65	21.31	71.98	71.87	317,197,054	83.00
WR52	91,892,853	13,174,543,743	34.47	15.59	45.45	45.21	266,474,097	69.73
WR81	96,647,340	13,826,809,815	36.18	25.00	69.15	69.09	274,917,517	71.94
WR103	95,866,473	13,859,437,006	46.92	46.70	46.87	46.7	269,178,161	70.44
WR111	83,267,631	11,884,028,952	31.10	9.61	31.14	30.91	260,345,610	68.13
WR133	72,753,336	10,352,724,630	27.09	18.49	68.33	68.26	270,764,352	70.85
WR 207	79,244,286	11,204,692,001	29.32	19.45	66.51	66.32	268,579,423	70.28
WR 37	77,206,124	11,323,300,556	29.63	8.96	30.52	30.25	257,716,289	67.44
WR 62	79,172,066	11,450,793,351	29.96	14.59	48.91	48.69	313,650,369	82.07
WR 100	79,922,587	11,616,872,760	30.40	15.44	51.00	50.78	266,834,329	69.82
WR126	60,113,679	8,704,460,829	22.78	12.54	55.23	55.04	263,722,779	69.01
WR 143	56,144,819	8,183,989,092	21.42	13.59	63.53	63.45	263,407,095	68.93
WR153	54,631,067	7,925,007,230	20.74	13.97	67.39	67.36	262,563,254	68.71
WR161	69,269,233	10,105,816,105	26.44	15.74	59.65	59.53	266,028,616	69.61
WR 171	75,698,012	11,057,490,330	28.93	11.06	38.45	38.21	258,379,810	67.61
WR184	65,726,306	9,580,839,457	25.07	13.44	53.82	53.61	262,299,170	68.64
WR195	65,675,525	9,450,951,461	24.73	15.50	62.78	62.66	267,892,702	70.10
WR219	84,313,296	12,275,734,508	32.12	21.77	67.83	67.76	269,530,363	70.53

WR230	75,856,818	11,038,295,474	28.88	20.48	70.93	70.89	268,379,182	70.23
WR233	75,798,242	10,936,594,201	28.62	9.25	32.47	32.31	257,764,957	67.45
WR 242	77,978,446	11,394,843,826	29.82	21.29	71.43	71.41	270,190,325	70.70
WR256	64,971,673	9,455,903,833	24.74	15.62	63.11	63.12	262,514,763	68.69
WR265	75,186,237	10,811,748,409	28.29	19.57	69.22	69.16	271,348,121	71.01
WR280	71,628,994	10,436,216,009	27.31	18.93	69.34	69.32	269,112,327	70.42
WR287	75,036,673	10,942,077,255	28.63	14.22	49.87	49.67	264,573,225	69.23

Table S2:Total number of single nucleotide polymorphisms (SNPs) and corresponding amino acid changes (AAC) of corresponding amino acid changes (AAC) of four seed shattering loci in 26 Australian wild rice samples: (A) *qSH1* (*Shattering (QTL)-1*); (B) *SH4*/*SHA1* (*SHATTERING 4*); (C) *SH1*/*OsSh1* (*SHATTERING 1*); (D) *SHAT1* (*SHATTERING ABORTION 1*)

locus	*qSH1*				*SH4*				*SH1/OsSh1*				*SHAT1*			
Sample	Heterozygous SNP	Heterozygous AAC	Homozygous SNP	Homozygous AAC	Heterozygous SNP	Heterozygous AAC	Homozygous SNP	Homozygous AAC	Heterozygous SNP	Heterozygous AAC	Homozygous SNP	Homozygous AAC	Heterozygous SNP	Heterozygous AAC	Homozygous SNP	Homozygous AAC
WR24	0	0	3	0	0	0	3	1	0	0	10	0	0	0	0	0
WR37	1	0	40	4	0	0	9	0	0	0	34	1	2	0	29	2
WR44	58	4	4	0	22	0	5	0	98	0	13	0	1	0	40	2
WR52	1	0	51	4	1	0	18	0	0	0	59	1	1	0	35	2
WR62	0	0	10	0	11	3	4	0	59	0	8	1	2	0	36	1
WR81	4	0	53	3	3	0	23	3	8	0	93	1	2	0	39	2
WR100	1	0	48	4	1	0	20	3	0	0	60	1	6	0	42	2
WR103	4	1	45	3	4	0	21	2	3	0	70	1	0	0	44	2
WR111	1	0	32	4	0	0	12	0	0	0	17	0	1	0	25	2
WR126	3	0	49	3	3	0	18	2	4	0	45	1	1	0	42	2
WR133	3	0	50	4	0	0	19	2	0	0	74	1	1	0	39	2
WR143	4	1	49	4	6	1	15	3	0	0	60	1	1	0	41	2
WR153	1	1	43	4	0	0	16	3	0	0	54	1	0	0	40	2
WR161	3	0	51	4	2	2	20	2	1	0	58	1	0	0	37	2
WR171	2	0	35	4	0	0	11	3	0	0	19	1	0	0	28	2
WR184	4	0	53	4	0	0	17	2	0	0	49	1	4	1	36	2
WR195	0	1	53	4	0	0	17	2	0	0	55	1	1	0	47	2
WR207	5	0	49	4	0	0	16	0	4	0	68	1	2	0	39	2
WR219	4	1	54	4	4	0	30	3	1	0	73	1	1	0	43	2
WR230	6	1	51	4	6	0	20	3	0	0	86	1	1	0	48	2
WR233	4	1	37	3	0	0	13	3	0	0	16	1	0	0	32	2
WR242	3	0	54	3	2	0	25	4	2	0	68	1	2	0	43	2

WR256	0	0	50	4	0	0	22	3	1	0	67	1	0	0	39	2
WR265	3	0	49	4	7	2	21	3	3	0	74	1	2	0	40	2
WR280	0	0	53	3	0	0	20	1	0	0	69	1	0	0	40	2
WR287	5	0	52	4	2	0	13	1	0	0	59	1	0	0	39	2

Table S3: List of non-synonymous amino acid changes in *qSH1* gene

Zygosity	Heterozygous			Homozygous		
Samples	No. of non-synonymous AAC	Amino acid change	Coding regions change	No. of non-synonymous AAC	Amino acid change	Coding regions change
WR37				4	Phe497Leu	1491C>A
					Ala358Gly	1073C>G
					Glu339Asp	1017G>C
					Pro101Ala	301C>G
WR44	4	Phe497Leu	1491C>A			
		Ala358Gly	1073C>G			
		Glu339Asp	1017G>C			
		Pro101Ala	301C>G			
WR52				4	Phe497Leu	1491C>A
					Ala358Gly	1073C>G
					Glu339Asp	1017G>C
					Pro101Ala	301C>G
WR81				3	Phe497Leu	1491C>A
					Ala358Gly	1073C>G
					Glu339Asp	1017G>C
WR100				4	Phe497Leu	1491C>A
					Ala358Gly	1073C>G
					Glu339Asp	1017G>C
					Pro101Ala	301C>G
WR103	1	His24Gln	72C>A	3	Phe497Leu	1491C>A
					Ala358Gly	1073C>G
					Glu339Asp	1017G>C
WR111				4	Phe497Leu	1491C>A

					Ala358Gly	1073C>G
					Glu339Asp	1017G>C
					Pro101Ala	301C>G
WR126				3	Phe497Leu	1491C>A
					Ala358Gly	1073C>G
					Glu339Asp	1017G>C
WR133				4	Phe497Leu	1491C>A
					Ala358Gly	1073C>G
					Glu339Asp	1017G>C
					Pro101Ala	301C>G
WR143	1	His24Gln	72C>A	4	Phe497Leu	1491C>A
					Ala358Gly	1073C>G
					Glu339Asp	1017G>C
					Pro101Ala	301C>G
WR153	1	His24Gln	72C>A	4	Phe497Leu	1491C>A
					Ala358Gly	1073C>G
					Glu339Asp	1017G>C
					Pro101Ala	301C>G
WR161				4	Phe497Leu	1491C>A
					Ala358Gly	1073C>G
					Glu339Asp	1017G>C
					Pro101Ala	301C>G
WR171				4	Phe497Leu	1491C>A
					Ala358Gly	1073C>G
					Glu339Asp	1017G>C
					Pro101Ala	*60G>C
WR184				4	Phe497Leu	1491C>A
					Ala358Gly	1073C>G
					Glu339Asp	1017G>C
					Pro101Ala	301C>G
WR195	1	His24Gln	72C>A	4	Phe497Leu	1491C>A

				Ala358Gly	1073C>G
				Glu339Asp	1017G>C
				Pro101Ala	301C>G
WR207			4	Phe497Leu	1491C>A
				Ala358Gly	1073C>G
				Glu339Asp	1017G>C
				Trp76Gly	226T>G
WR219	His24Gln	72C>A	4	Phe497Leu	1491C>A
				Ala358Gly	1073C>G
				Glu339Asp	1017G>C
				Pro101Ala	301C>G
WR230	His24Gln	72C>A	3	Phe497Leu	1491C>A
				Ala358Gly	1073C>G
				Glu339Asp	1017G>C
WR233	His24Gln	72C>A	3	Phe497Leu	1491C>A
				Ala358Gly	1073C>G
				Glu339Asp	1017G>C
WR242			3	Ala358Gly	1073C>G
				Glu339Asp	1017G>C
				Pro101Ala	301C>G
WR256			4	Phe497Leu	1491C>A
				Ala358Gly	1073C>G
				Glu339Asp	1017G>C
				Arg110Cys	328C>T
WR265			4	Phe497Leu	1491C>A
				Ala358Gly	1073C>G
				Glu339Asp	1017G>C
				Phe24Leu	70T>C
WR280			3	Phe497Leu	1491C>A
				Ala358Gly	1073C>G
				Glu339Asp	1017G>C

WR287	4	Phe497Leu	1491C>A
		Ala358Gly	1073C>G
		Glu339Asp	1017G>C
		Pro101Ala	301C>G

Table S4: List of non-synonymous amino acid changes in *SH4* gene

Zygosity	Heterozygous			Homozygous		
Samples ID	No. of non-synonymous AAC	Amino acid change	Coding regions change	No. of non-synonymous AAC	Amino acid change	Coding regions change
WR24				1	Gly297Asp	890G>A
WR62	3	Gln243His	729G>C			
		Ala167Val	500C>T			
		Thr158Ala	472A>G			
WR81				3	Ala167Val	500C>T
					**Asn79Lys**	**237T>G**
					Val19Ala	56T>C
WR100				3	Ala167Val	500C>T
					Thr158Ala	472A>G
					**Asn79Lys**	**237T>G**
WR103				2	Thr158Ala	472A>G
					**Asn79Lys**	**237T>G**
WR126				2	Ala167Val	500C>T
					**Asn79Lys**	**237T>G**
WR133				2	Ala167Val	500C>T
					Thr158Ala	472A>G
WR143	1	Leu226Pro	677T>C	3	Ala167Val	500C>T
					Thr158Ala	472A>G
					**Asn79Lys**	**237T>G**
WR153				3	Ala167Val	500C>T
					Thr158Ala	472A>G
					**Asn79Lys**	**237T>G**
WR161	2	Ala155Ser	463G>T	2	Thr158Ala	472A>G
		Val152Ala	455T>C		**Asn79Lys**	**237T>G**
WR171				3	Ala167Val	500C>T

					Thr158Ala	472A>G
					**Asn79Lys**	**237T>G**
WR184				2	Ala167Val	500C>T
					Thr158Ala	472A>G
WR195				2	Thr158Ala	472A>G
					Val19Ala	56T>C
WR219				3	Thr158Ala	472A>G
					**Asn79Lys**	**237T>G**
					Val19Ala	56T>C
WR230				3	Ala167Val	500C>T
					Thr158Ala	472A>G
					**Asn79Lys**	**237T>G**
WR233				3	Ala167Val	500C>T
					Thr158Ala	472A>G
					Asn79Lys	237T>G
WR242				4	Ala167Val	500C>T
					Thr158Ala	472A>G
					**Asn79Lys**	**237T>G**
					Val19Ala	56T>C
WR256				3	Ala167Val	500C>T
					Thr158Ala	472A>G
					**Asn79Lys**	**237T>G**
WR265	2	Leu226Pro	677T>C	3	Ala167Val	500C>T
		Ala155Ser	463G>T		Thr158Ala	472A>G
					Val19Ala	56T>C
WR280				1	**Asn79Lys**	**237T>G**
WR287				1	Thr158Ala	472A>G

Table S5: Total number of single nucleotide polymorphisms (SNPs) and corresponding amino acid changes (AAC) of corresponding amino acid changes (AAC) of four awn development loci in 26 Australian wild rice samples: (A) *AN3-1* (*Awn3-1*); (B) *LABA1* (*LONG AWN AND BARB 1*); (C) *RAE1* (*REGULATION OF AWN ELONGATION 1*); (D) *RAE2* (*REGULATION OF AWN ELONGATION 2*)

Locus	*AN3-1*				*LABA1*				*RAE1*				*RAE2*			
Sample	Heterozygous SNP	Heterozygous AAC	Homozygous SNP	Homozygous AAC	Heterozygous SNP	Heterozygous AAC	Homozygous SNP	Homozygous AAC	Heterozygous SNP	Heterozygous AAC	Homozygous SNP	Homozygous AAC	Heterozygous SNP	Heterozygous AAC	Homozygous SNP	Homozygous AAC
WR24	0	0	0	0	0	0	0	0	19	4	3	1	0	0	1	0
WR37	17	5	28	3	0		20	0	1	0	31	6	0	0	5	2
WR44	2	1	5	1	0	0	59	0	52	8	5	2	11	2	1	0
WR52	18	3	30	2	0	0	53	0	0	0	37	7	1	0	12	2
WR62	15	0	23	3	39	0	1	0	39	6	6	2	14	2	1	0
WR81	19	5	36	3	1	0	61	0	0	0	59	11	5	0	18	2
WR100	20	5	29	2	0	0	48	0	0	0	51	10	3	0	12	2
WR103	24	5	28	3	0	0	57	0	0	0	51	7	4	0	15	2
WR111	15	5	26	3	0	0	28	0	0	0	23	5	3	0	4	2
WR126	15	2	26	2	5	0	50	0	4	1	27	5	5	0	12	2
WR133	19	5	30	3	1	0	59	0	0	0	49	12	4	0	13	2
WR143	15	4	30	3	0	0	40	0	0	0	33	8	0	0	15	2
WR153	14	5	25	3	0	0	32	0	0	0	57	13	3	0	13	2
WR161	20	4	28	3	1	0	55	0	0	0	52	10	3	0	16	2
WR171	21	4	21	2	0	0	24	0	0	0	21	8	0	0	7	2
WR184	31	7	29	3	0	0	40	0	0	0	27	4	3	0	7	1
WR195	22	4	28	4	0	0	45	0	0	0	54	11	4	0	19	2
WR207	18	3	30	2	0	0	51	0	0	0	45	9	2	0	12	2
WR219	23	8	32	1	0	0	53	1	0	0	49	7	4	0	15	2
WR230	24	6	30	2	0	0	55	0	0	0	53	11	0	0	15	2
WR233	17	5	17	3	0	0	15	0	0	0	14	3	0	0	3	2
WR242	18	5	24	1	1	0	58	0	0	0	51	9	3	0	18	2
WR256	20	4	23	3	1	0	52	0	0	0	50	11	4	0	13	2

WR265	16	5	31	3	1	0	54	0	0	0	50	10	3	0	13	2
WR280	11	4	28	3	1	0	51	0	0	0	44	8	3	0	18	2
WR287	16	4	23	3	0	0	56	0	0	0	48	9	1	0	9	2

Table S6: List of non-synonymous amino acid changes in *AN3-1* gene

Zygosity	Heterozygous			Homozygous		
Samples ID	No. of non-synonymous AAC	Amino acid change	Coding regions change	No. of non-synonymous AAC	Amino acid change	Coding regions change
WR37	5	Ala134Val	401C>T	3	Thr121Ala	361A>G
		Ala128Val	383C>T		Met111Thr	332T>C
		Gln115Arg	344A>G		His104Arg	311A>G
		Ala108Thr	322G>A			
		Pro96Ser	286C>T			
WR44	1	Gln115Arg	344A>G	2	Thr121Ala	361A>G
WR52	3	Ala146Thr	436G>A	2	Thr121Ala	361A>G
		Gln115Arg	344A>G		Met111Thr	332T>C
		His104Arg	311A>G			
WR62				3	Thr121Ala	361A>G
					Gln115Arg	344A>G
					Met111Thr	332T>C
WR81	5	Ala146Thr	436G>A	3	Thr121Ala	361A>G
		Ala134Val	401C>T		Met111Thr	332T>C
		Gln115Arg	344A>G		His104Arg	311A>G
		Arg105*	313C>T			
		Arg82Lys	245G>A			
WR100	5	Ala134Val	401C>T	2	Thr121Ala	361A>G
		Gln115Arg	344A>G		Met111Thr	332T>C
		His104Arg	311A>G			
		Arg82Lys	245G>A			
		Arg76His	227G>A			
WR103	5	Ala134Val	401C>T	3	Thr121Ala	361A>G
		Gln115Arg	344A>G		Met111Thr	332T>C
		Ala108Thr	322G>A		His104Arg	311A>G
		Pro96Ser	286C>T			
		Arg92His	275G>A			
WR111	5	Ala146Thr	436G>A	3	Thr121Ala	361A>G
		Ala134Val	401C>T		Met111Thr	332T>C
		Gln115Arg	344A>G		His104Arg	311A>G

		Ala108Thr	322G>A			
		Pro96Ser	286C>T			
WR126	2	Ala134Val	401C>T	2	Thr121Ala	361A>G
		His104Arg	311A>G		Met111Thr	332T>C
WR133	5	Ala146Thr	436G>A	3	Thr121Ala	361A>G
		Ala134Val	401C>T		Met111Thr	332T>C
		Gln115Arg	344A>G		His104Arg	311A>G
		Ala108Thr	322G>A			
		Pro96Ser	286C>T			
WR143	4	Ala146Thr	436G>A	3	Thr121Ala	361A>G
		Ala134Val	401C>T		Met111Thr	332T>C
		Gln115Arg	344A>G		His104Arg	311A>G
		Arg105*	313C>T			
WR153	5	Ala146Thr	436G>A	3	Thr121Ala	361A>G
		Ala134Val	401C>T		Met111Thr	332T>C
		Gln115Arg	344A>G		His104Arg	311A>G
		Arg105*	313C>T			
		Arg76His	227G>A			
WR161	4	Ala134Val	401C>T	3	Thr121Ala	361A>G
		Ala128Val	383C>T		Met111Thr	332T>C
		Gln115Arg	344A>G		His104Arg	311A>G
		Ala108Thr	322G>A			
WR171	4	Ala134Val	401C>T	2	Met111Thr	332T>C
		Thr121Ala	361A>G		His104Arg	311A>G
		Gln115Arg	344A>G			
		Pro114Ser	340C>T			
WR184	7	Ala146Thr	436G>A	3	Thr121Ala	361A>G
		Ala134Val	401C>T		Met111Thr	332T>C
		Gln115Arg	344A>G		His104Arg	311A>G
		Arg105*	313C>T			
		Arg92His	275G>A			
		Arg82Lys	245G>A			
		Arg76His	227G>A			
WR195	4	Ala146Thr	436G>A	4	Thr121Ala	361A>G
		Gln115Arg	344A>G		Met111Thr	332T>C
		Ala108Thr	322G>A		His104Arg	311A>G
		Pro96Ser	286C>T		Arg85His	254G>A
WR207	3	Ala146Thr	436G>A	2	Thr121Ala	361A>G
		Gln115Arg	344A>G		Met111Thr	332T>C

		His104Arg	311A>G			
WR219	8	Ala134Val	401C>T	1	His104Arg	311A>G
		Thr121Ala	361A>G			
		Gln115Arg	344A>G			
		Pro114Ser	340C>T			
		Gly87Arg	259G>A			
		Arg76His	227G>A			
		Arg73Cys	217C>T			
		Pro72Leu	215C>T			
WR230	6	Ala134Val	401C>T	2	Thr121Ala	361A>G
		Ala108Thr	322G>A		Met111Thr	332T>C
		His104Arg	311A>G			
		Arg91Cys	271C>T			
		Arg85His	254G>A			
		Arg82Lys	245G>A			
WR233	5	Ala146Thr	436G>A	3	Thr121Ala	361A>G
		Ala134Val	401C>T		Met111Thr	332T>C
		Gln115Arg	344A>G		His104Arg	311A>G
		Ala108Thr	322G>A			
		Pro96Ser	286C>T			
WR242	5	Ala134Val	401C>T	1	Thr121Ala	361A>G
		Gln115Arg	344A>G			
		His104Arg	311A>G			
		Arg82Lys	245G>A			
		Arg76His	227G>A			
WR256	4	Ala146Thr	436G>A	3	Thr121Ala	361A>G
		Ala134Val	401C>T		Met111Thr	332T>C
		Gln115Arg	344A>G		His104Arg	311A>G
		Ala108Thr	322G>A			
WR265	5	Ala146Thr	436G>A	3	Thr121Ala	361A>G
		Ala134Val	401C>T		Met111Thr	332T>C
		Gln115Arg	344A>G		His104Arg	311A>G
		Ala108Thr	322G>A			
		Pro96Ser	286C>T			
WR280	4	Ala134Val	401C>T	3	Thr121Ala	361A>G
		Gln115Arg	344A>G		Met111Thr	332T>C
		Ala108Thr	322G>A		His104Arg	311A>G
		Pro96Ser	286C>T			
WR287	4	Ala146Thr	436G>A	3	Thr121Ala	361A>G

	Ala134Val	401C>T	Met111Thr	332T>C
	Ala128Val	383C>T	His104Arg	311A>G
	Ala108Thr	322G>A		

Table S7: List of non-synonymous amino acid changes in *RAE1* gene

Zygosity	Heterozygous			Homozygous		
Samples	No. of non-synonymous AAC	Amino acid change	Coding regions change	No. of non-synonymous AAC	Amino acid change	Coding regions change
WR24	4	Asp77Gly	230A>G	1	Ala63Gly	188C>G
WR37				6	Ser178Arg	534C>G
					Ser178Gly	532A>G
					Asp77Gly	230A>G
					Ala68Glu	203C>A
					Ala63Gly	188C>G
					Asp59Glu	177C>A
WR44	8	Thr254Ala	760A>G	2	Ser178Gly	532A>G
		Val200Ala	599T>C		Ala63Gly	188C>G
		Thr199Ala	595A>G			
		Ser178Arg	534C>G			
		Asp77Gly	230A>G			
		Ala68Glu	203C>A			
		Asp66Gly	197A>G			
		Asp59Glu	177C>A			
WR52				7	Thr254Ala	760A>G
					Ser178Arg	534C>G
					Ser178Gly	532A>G
					Asp77Gly	230A>G
					Asp66Gly	197A>G
					Ala63Gly	188C>G
					Asp59Glu	177C>A
WR62	6	Thr254Ala	760A>G	2	Ser178Gly	532A>G
		Ser178Arg	534C>G		Ala63Gly	188C>G
		Asp77Gly	230A>G			

	Ala68Glu	203C>A			
	Asp66Gly	197A>G			
	Asp59Glu	177C>A			
WR81			11	Pro241Ser	721C>T
				Ala227Thr	679G>A
				Val200Ala	599T>C
				Thr199Ala	595A>G
				Ser178Arg	534C>G
				Ser178Gly	532A>G
				Asp77Gly	230A>G
				Ala68Glu	203C>A
				Asp66Gly	197A>G
				Ala63Gly	188C>G
				Asp59Glu	177C>A
WR100			10	Thr254Ala	760A>G
				Pro241Ser	721C>T
				Ala227Thr	679G>A
				Ser178Arg	534C>G
				Ser178Gly	532A>G
				Asp77Gly	230A>G
				Ala68Glu	203C>A
				Asp66Gly	197A>G
				Ala63Gly	188C>G
				Asp59Glu	177C>A
WR103			7	Pro241Ser	721C>T
				Ala227Thr	679G>A
				Ser178Arg	534C>G
				Ser178Gly	532A>G
				Asp77Gly	230A>G
				Ala63Gly	188C>G
				Asp59Glu	177C>A

WR111				5	Ser178Arg	534C>G
					Phe157Ile	469T>A
					Asp77Gly	230A>G
					Ala68Glu	203C>A
WR126	1	Phe157Ile	469T>A	5	Thr254Ala	760A>G
					Ser178Arg	534C>G
					Ser178Gly	532A>G
					Ala63Gly	188C>G
					Asp59Glu	177C>A
WR133				12	Thr254Ala	760A>G
					Val200Ala	599T>C
					Thr199Ala	595A>G
					Thr194Ala	580A>G
					Ala185Thr	553G>A
					Ser178Arg	534C>G
					Ser178Gly	532A>G
					Asp77Gly	230A>G
					Ala68Glu	203C>A
					Asp66Gly	197A>G
					Ala63Gly	188C>G
					Asp59Glu	177C>A
WR143				8	Thr254Ala	760A>G
					Ser178Arg	534C>G
					Ser178Gly	532A>G
					Asp77Gly	230A>G
					Ala68Glu	203C>A
					Asp66Gly	197A>G
					Ala63Gly	188C>G
					Asp59Glu	177C>A
WR153				13	Thr254Ala	760A>G
					Phe245Tyr	734T>A

		Ala227Thr	679G>A
		Val200Ala	599T>C
		Thr199Ala	595A>G
		Thr194Ala	580A>G
		Ser178Arg	534C>G
		Ser178Gly	532A>G
		Asp77Gly	230A>G
		Ala68Glu	203C>A
		Asp66Gly	197A>G
		Ala63Gly	188C>G
		Asp59Glu	177C>A
WR161	10	Thr254Ala	760A>G
		Pro241Ser	721C>T
		Ala227Thr	679G>A
		Val200Ala	599T>C
		Thr199Ala	595A>G
		Ser178Arg	534C>G
		Ser178Gly	532A>G
		Asp77Gly	230A>G
		Ala63Gly	188C>G
		Asp59Glu	177C>A
WR171	8	Thr254Ala	760A>G
		Ser178Arg	534C>G
		Ser178Gly	532A>G
		Asp77Gly	230A>G
		Ala68Glu	203C>A
		Asp66Gly	197A>G
		Ala63Gly	188C>G
		Asp59Glu	177C>A
WR184	4	Thr254Ala	760A>G
		Asp77Gly	230A>G

		Ala63Gly	188C>G
		Asp59Glu	177C>A
WR195	11	Thr254Ala	760A>G
		Ala227Thr	679G>A
		Ala216Val	647C>T
		Ser178Arg	534C>G
		Ser178Gly	532A>G
		Phe157Ile	469T>A
		Asp77Gly	230A>G
		Ala68Glu	203C>A
		Asp66Gly	197A>G
		Ala63Gly	188C>G
		Asp59Glu	177C>A
WR207	9	Thr254Ala	760A>G
		Pro241Ser	721C>T
		Ala227Thr	679G>A
		Ser178Arg	534C>G
		Ser178Gly	532A>G
		Asp77Gly	230A>G
		Ala68Glu	203C>A
		Ala63Gly	188C>G
		Asp59Glu	177C>A
WR219	7	Thr254Ala	760A>G
		Ala216Val	647C>T
		Ser178Arg	534C>G
		Ser178Gly	532A>G
		Asp77Gly	230A>G
		Ala63Gly	188C>G
		Asp59Glu	177C>A
WR230	11	Thr254Ala	760A>G
		Ala227Thr	679G>A

		Ala216Val	647C>T
		Val200Ala	599T>C
		Thr194Ala	580A>G
		Ser178Arg	534C>G
		Ser178Gly	532A>G
		Phe157Ile	469T>A
		Asp77Gly	230A>G
		Ala63Gly	188C>G
		Asp59Glu	177C>A
WR233	3	Asp77Gly	230A>G
		Ala63Gly	188C>G
		Asp59Glu	177C>A
WR242	9	Thr254Ala	760A>G
		Pro241Ser	721C>T
		Ala227Thr	679G>A
		Ala216Val	647C>T
		Ser178Arg	534C>G
		Ser178Gly	532A>G
		Asp77Gly	230A>G
		Ala63Gly	188C>G
		Asp59Glu	177C>A
WR256	11	Thr254Ala	760A>G
		Ala227Thr	679G>A
		Ala216Val	647C>T
		Val200Ala	599T>C
		Thr194Ala	580A>G
		Ser178Arg	534C>G
		Ser178Gly	532A>G
		Phe157Ile	469T>A
		Asp77Gly	230A>G
		Ala63Gly	188C>G

		Asp59Glu	177C>A
WR265	10	Pro241Ser	721C>T
		Val200Ala	599T>C
		Thr199Ala	595A>G
		Ser178Arg	534C>G
		Ser178Gly	532A>G
		Asp77Gly	230A>G
		Ala68Glu	203C>A
		Asp66Gly	197A>G
		Ala63Gly	188C>G
		Asp59Glu	177C>A
WR280	8	Thr254Ala	760A>G
		Pro241Ser	721C>T
		Ala227Thr	679G>A
		Ser178Arg	534C>G
		Ser178Gly	532A>G
		Asp77Gly	230A>G
		Ala63Gly	188C>G
		Asp59Glu	177C>A
WR287	9	Thr254Ala	760A>G
		Ala227Thr	679G>A
		Ala216Val	647C>T
		Val200Ala	599T>C
		Thr194Ala	580A>G
		Ser178Arg	534C>G
		Ser178Gly	532A>G
		Phe157Ile	469T>A
		Asp77Gly	230A>G

Table S8: Total number of single nucleotide polymorphisms (SNPs) and corresponding amino acid changes (AAC) of three grain size loci in 26 Australian wild rice samples: (A) *GS2* (*GRAIN SIZE 2*); (B) *GS5* (*GRAIN SIZE 5*); (C) *GW8* (*GRAIN WIDTH 8*)

Locus	*GS2*				*GS5*				*GW8*			
Sample	Heterozygous SNP	Heterozygous AAC	Homozygous SNP	Homozygous AAC	Heterozygous SNP	Heterozygous AAC	Homozygous SNP	Homozygous AAC	Heterozygous SNP	Heterozygous AAC	Homozygous SNV	Homozygous AAC
WR24	0	0	6	0	1	0	1	1	0	0	0	0
WR37	0	0	46	5	0	0	4	1	1	0	42	3
WR44	0	0	13	1	0	0	6	0	56	3	2	0
WR52	0	0	68	5	0	0	5	0	0	0	40	2
WR62	48	6	1	0	0	0	4	0	0	0	9	1
WR81	0	0	70	5	0	0	7	1	0	0	57	2
WR100	0	0	63	5	0	0	7	1	0	0	46	2
WR103	0	0	66	5	0	0	7	1	0	0	41	2
WR111	0	0	40	5	1	0	2	0	0	0	19	1
WR126	0	0	50	5	2	0	6	1	0	0	30	2
WR133	0	0	65	5	0	0	6	1	0	0	47	2
WR143	0	0	54	5	0	0	4	1	1	1	37	2
WR153	0	0	54	5	0	0	4	2	1	0	34	2
WR161	0	0	67	5	0	0	6	1	0	0	44	2
WR171	0	0	35	5	0	0	1	0	0	0	19	2
WR184	0	0	57	5	0	0	6	1	0	0	40	2
WR195	0	0	65	5	0	0	5	1	0	0	35	2
WR207	0	0	71	6	0	0	71	3	4	2	47	2
WR219	0	0	71	5	0	0	6	1	0	0	54	1
WR230	0	0	60	5	0	0	5	1	0	0	48	2
WR233	0	0	29	1	0	0	2	1	1	1	22	1
WR242	0	0	64	5	0	0	5	1	0	0	46	2
WR256	0	0	59	5	0	0	7	1	0	0	42	2
WR265	0	0	64	6	0	0	6	1	0	0	49	2
WR280	0	0	59	6	0	0	6	1	0	0	47	2

WR287	0	0	57	5	1	0	6	1	3	0	39	2

Table S9: Summary of 26 wild rice samples collected from 26 different geographical locations in the norther Queensland, Australia

Samples ID	Species	Site	GPS coordinates
WR24	*Oryza rufipogon type taxa*	Abbatoir Swamp (Mosman Mount Moloy Road)	S:16.63574° E:145.32603°
WR44	Hybrid	Lakeland Cook Town Road Mulligan Hwy (White lily lake)	S:15.758640° E: 144.99924°
WR52	Hybrid	Lakeland Cook Town Road (White lily lake)	S:15.758640° E:144.99924°
WR81	*Oryza meridionalis*	Meridionalis Site	S: 15.53078° E: 144.38336°
WR103	*Oryza meridionalis*	Lakefield National Park Site-2 (Ditch near White lily Lagoon)	S: 14.85996° E: 144.16586°
WR111	*Oryza meridionalis*	Red Lilly Lagoon JPN2 Type (JPN11-site Ryuji)	S: 14.84947° E: 144.16811°
WR133	*Oryza meridionalis*	JPN2 site (Small lake)	S: 15.43943° E: 144.21111°
WR207	*Oryza meridionalis*	Telegraph Road (Weipa turnoff to Bata via Downs)	S: 12.88274° E: 142.73929°
WR37	*Oryza meridionalis*	Brooklyn National Park Mulligan Hwy (outside across the road)	S:16.57874° E: 145.18906°
WR62	Hybrid	Lakeland Cook Town Road (White lily lake)	S:15.758640° E: 144.99924°
WR100	*Oryza meridionalis*	Lakefield National Park site-2	S: 14.85996° E: 144.16586°
WR126	*Oryza meridionalis*	Lakefield National Park site-3	S; 15.14672° E: 144.32773

WR143	*Oryza meridionalis*	Balurga Road Site-1 (off Musgrave to pormpurraw road)	S: 14.83915° E: 142.56808°
WR153	Hybrid	Balurga Road Site-2(off Musgrave to pormpurraw road)	S: 14.90241° E: 142.49919°
WR161	Hybrid	Merluna Site-1 (lake)	S: 13.05811° E: 142.61964°
WR171	*Oryza meridionalis*	Andoom Road in Weipa (wetland)	S: 12.61513° E: 141.89191°
WR184	*Oryza meridionalis*	Andoom Road in Weipa (wetland)	S: 12.66010° E: 142.66843°
WR195	*Oryza meridionalis*	Development road to Bamaga SITE-1	S: 12.45885° E: 142.63562°
WR219	*Oryza meridionalis*	peninsular Development Road Site-1	S: 13.29167° E: 142.84729°
WR230	*Oryza meridionalis*	Peninsular Development Road Site-3	S: 14.005117° E: 143.19036°
WR233	*Oryza meridionalis*	peninsular Development Road Site-4	S: 14.785617° E: 143.50446°
WR242	*Oryza meridionalis*	Peninsular Development Road Site-5	S: 15.00745° E: 143.64099°
WR256	*Oryza meridionalis*	Townsville site 1 (Bruce Highway 30 km south Townsville)	S:19.395962 E: 147.004486
WR265	*Oryza meridionalis*	Townsville site-2 (Woodstock Giru Road)	S:19.599657 E: 146.882965
WR280	*Oryza meridionalis*	Townsville site-3 (Charters Towers Townsville road)	S:19.397224 E: 146.723831
WR287	*Oryza meridionalis*	Townsville site-4 (Town Common wetlands Townsville)	S:19.25445 E: 146.725586

Table S10: Sequences of domestication genes analyzed. Sequences of domestication loci were extracted from the rice reference genome (Os-Nipponbare-Reference-IRGSP-1.0) downloaded at Rice Annotation Project Database (RAP-DB) [46].

Gene Symbol	Gene Name	Gene ID	Chromosome	Type	Region		Function
					start base position	end base position	
qSH1	*Shattering (QTL)-1*	Os01g0848400	chr01	gene	36445456	36449951	Initiation and maintenance of the shoot apical meristem during embryogenesis
SH4	*SHATTERING 4*	Os04g0670900	chr04	gene	34231186	34233221	Control of the abscission layer formation
OsSH1	*SHATTERING 1*	Os03g0650000	chr03	gene	25197057	25206948	YABBY transcription factor, Control of seed shattering
SHAT1	*SHATTERING ABORTION 1*	Os04g0649100	chr04	gene	33071622	33075371	Seed shattering through abscission zone (AZ) development
AN3-1	*AWN3-1*	Os03g0418600	chr03	gene	17410000	17412951	Control of awn development
LABA1	*LONG AND BARBED AWN 1*	Os04g0518800	chr04	gene	25959399	25963504	Cytokinin synthesis enzyme, Regulation of awn length and grain production
RAE1	*REGULATOR OF AWN ELONGATION 1*	Os04g0350700	chr04	gene	16731738	16735336	Basic helix-loop-helix protein, Regulation of awn development, grain size, and grain number
RAE2	*REGULATOR OF AWN ELONGATION 2*	Os08g0485500	chr08	gene	23998787	24000176	Epidermal patterning factor-like 1 (EPFL1) protein, Regulation of grain number, grain length, and awn development (Dysfunctional RAE2 protein)
GS2	*GRAIN SIZE 2*	Os02g0701300	chr02	gene	28863274	28866997	Growth-regulating factor, Regulation of grain shape and panicle length
GS5	*GRAIN SIZE 5*	Os05g0158500	chr05	gene	3439304	3443769	Serine carboxypeptidase, Positive regulator of grain size
GW8	*GRAIN-WIDTH 8*	Os08g0531600	chr08	gene	26501167	26506198	Transcription factor, Positive regulator of cell proliferation, Control of grain size, shape, and quality
